# Chloride of Zinc

**Published:** 1884-02

**Authors:** 


### Editorial.

### CHLORIDE OF ZINC.

The Professor of Special Surgery to whose lectures we listened in college days, had less faith in the virtues of medicines than most men. He was accustomed to say in his peculiar, drawling, but sententious manner: "Gentlemen, when you enter upon practice don't multiply remedies. If a cure of disease was ever wrought directly by medicine, it was probably done by iodide of potassium in a case of syphilis. If I were to order a new medicine case, I should, I think, have it in five bottles. In the first I would place—morphia; in the second—atropia; in the third—iodide of potassium; in the fourth —well, I think I would put in a little more iodide of potassium; in the fifth ——gentlemen, I have made the case too large. I don't know what I could put in the fifth bottle."

For most of the cases that fall under the care of the average dentist, we are strongly inclined to give to chloride of zinc the same prominence that the old Professor gave to iodide of potassium. Its action is so beneficent, though at times somewhat severe, that we would advise the young practitioner to put into the empty bottle "a little more chloride of zinc."

It is prepared by disolving zinc, or its oxide or carbonate, in hydrochloric acid, and then filtering the solution, and evaporating it to dryness. It is a grayish-white, semi-transparent, and gelatinous substance, but if fully dried becomes solid and pulverizable. It deliquesces on exposure to the air, is soluble in water, alcohol, and ether, and unites with both albumen and gelatine. It has an acid and metallic taste (Stillé). It is a corrosive agent, but differs from most such by its absence of mischevious effects. It is not readily absorbed, and if it be, it will do no injury. As a topical application it is astringent, it is a deodorizer and a disinfectant, but its principal virtue is its astonishing power in promoting healthy granulations in stagnating tissues. In all cases of chronic inflammation of the gums, in pyorrhea alveolaris, and as a dressing for indolent wounds after surgical operations, we have found it extremely useful. As a topical application about the gingival margins after the removal of tartar, it is invaluable. In the deep pockets beneath the gum, caused by calcareous or other deposits, that are not of a catarrhal character, we have found nothing to quite take its place.

In the condition now commonly denominated pyorrhea alveolaris, when there is denudation and separation of the gum from the teeth, an absorption or destruction of the alveolar border so that a probe can be thrust for a greater or less distance under the gum, with a constant discharge of pus, we have not infrequently brought about a cure by the use of chloride of zinc alone. It is better, however, to give surgical assistance first, by scraping the edges of the process, and this may profitably be followed by the use of thorough antiseptics until the discharge is stopped. At this point, if all foreign accumulations be removed from the tooth, there is no dressing that has yielded better results for the after treatment than chloride of zinc. The gum about the cervical portion of the tooth in such cases is almost invariably turgid and swollen, so that to the casual observer there would seem to be no apparent denudation; but when the inflammation has subsided it will be found that the tissue shrinks away sufficiently to expose a considerable portion of the root. If this tumefied tissue be too thoroughly cauterized, there will be an entire destruction of that which under more judicious treatment might possibly be retained and restored to normal function. The principal aim of the dentist in such cases, should be to retain as much of a "pocket" as possible, for without something of this kind there can be no restoration of that which has been lost. When the breaking down of tissue has ceased, and diseased action has reached a limit, the process of repair should at once commence, but this cannot be secured without something to stand as a protector for the protoplasmic matter which may be deposited. New granulations cannot be added unless they are secured from foreign interference. Hence the necessity for the preservation of the sheltering gum.

Chloride of zinc in dilution, is sufficiently escharotic to cauterize the inflamed surfaces without destroying them. It is such a powerful stimulant that, if all irritating causes be removed, new granulations almost invariably succeed its employment.

In pyorrhea alveolaris, after the removal of all deposits, the edges of the diseased alveolus may be thoroughly saturated with aromatic sulphuric acid, which will not attack the soft tissues, and the case allowed to rest for a few days. If then the discharge has ceased, the treatment with chloride of zinc may be commenced. If there still remains any trace of discharge, it is an indication that some of the diseased parts have not been reached, and a thorough exploration should again be made for deposits and diseased bone, and the sulphuric acid once more be resorted to. But if there be no longer any discharge, a solution of chloride of zinc, of perhaps ten grains to the ounce of water, may be injected beneath the gums, and this repeated two or three times a week, until a cure has been effected. A little experience will soon teach one the proper strength of the solution to be used. Some cases demand more severe treatment than others. A special syringe should be kept for the use of this remedy, and that the point may not become obstructed, it may be allowed to remain point downward in a glass partly filled with water.

Chloride of zinc is very useful in catarrhal conditions of the maxillary sinus, or antrum of Highmore. It will be necessary, however, to use care in its employment, and to inject it considerably dilute, as intense pain follows the use of a solution too concentrated. One of the most obstinate cases of ulceration of the mucous membrane of this sinus which ever come under our care, yielded to a solution of about twenty grains to the ounce, after almost everything else had failed, but the patient will not soon forget its application. One dose was enough for both him and the disease.

Chloride of zinc is also one of the best obtundents of sensitive dentine that we possess, although its first application is liable to be irritating. A drop of the deliquescent fluid is placed in the cavity, which has previously been thoroughly dried, and allowed to remain for four or five minutes. When the obtunded layer of dentine shall have been removed, a fresh application will be necessary.

